# Vaccination as a risk factor for pediatric multiple sclerosis: Insights from a retrospective case–control study

**DOI:** 10.1177/13524585241297003

**Published:** 2024-11-14

**Authors:** Sonia Darvishi, Ewan Donnachie, Christiane Gasperi, Alexander Hapfelmeier, Bernhard Hemmer

**Affiliations:** Department of Neurology, Klinikum rechts der Isar, School of Medicine and Health, Technical University of Munich, Munich, Germany; Bavarian Association of Statutory Health Insurance Physicians, Munich, Germany; Department of Neurology, Klinikum rechts der Isar, School of Medicine and Health, Technical University of Munich, Munich, Germany; Institute of General Practice and Health Services Research, School of Medicine and Health, Technical University of Munich, Munich, Germany; Institute of AI and Informatics in Medicine, School of Medicine and Health, Technical University of Munich, Munich, Germany; Department of Neurology, Klinikum rechts der Isar, School of Medicine and Health, Technical University of Munich, Munich, Germany; Munich Cluster for Systems Neurology (SyNergy), Munich, Germany

**Keywords:** Pediatric multiple sclerosis, vaccination, diagnosis

## Abstract

This study evaluated the association between pediatric multiple sclerosis and vaccinations within 5 years before diagnosis using German ambulatory claims data. Children with multiple sclerosis (*n* = 346) aged 9–17 were analyzed with logistic and Poisson regression. Control groups included children with Crohn’s disease, psoriasis, and no autoimmune diseases. The results indicated a negative association between vaccinations and pediatric multiple sclerosis, with no significant risk identified. This negative relationship was consistent in sensitivity and clinically isolated syndrome analyses. Overall, the study’s findings do not support the hypothesis that vaccination is a risk factor for pediatric multiple sclerosis.

## Introduction

Pediatric multiple sclerosis (MS) is defined as a disease onset of MS before the age of 18.^1^ The etiology of MS is multifactorial, involving genetic predisposition and environmental factors.^2–4^ The potential association between childhood vaccination and the subsequent development of MS has been a subject of considerable debate and research.^5,6^ Given the critical role of vaccinations in preventing many infectious diseases, it is essential to rigorously assess their safety profile concerning autoimmune diseases such as MS.

We performed a population-based retrospective cohort study to investigate whether childhood vaccination could be a risk factor for the onset of pediatric MS, using three control cohorts for comparison.

## Methods

We conducted a retrospective case–control study using ambulatory claims data from the Bavarian Association of Statutory Health Insurance Physicians (BASHIP) covering 2005 to 2020. BASHIP’s ambulatory claims data cover all the approximately 11 million members of Bavaria’s statutory health insurance, which accounts for about 85% of the population. It includes diagnoses coded according to the German version of the International Classification of Diseases (10th edition, ICD-10) and information about vaccinations by service records of quarterly reimbursement claims, sex, and age. Data were available for 2005 to 2020. Since vaccines are also administered in combination, we classified them into ten vaccine groups: (1) tick-borne encephalitis (TBE), (2) hepatitis A, (3) hepatitis B, (4) influenza virus, (5) meningococci, (6) MMR and VZV (MMRV), (7) pneumococci, (8) *Clostridium tetani, Corynebacterium diphtheriae*, poliovirus, *Bordetella pertussis*, and Hemophilus influenza type B (DPTPH), (9) human papillomavirus (HPV) and (10) any vaccination. For members of the statutory health insurance, the listed vaccinations are free of charge since they are part of the set of vaccines recommended by the German Standing Committee on Vaccination.

We defined a cohort of children diagnosed with pediatric MS (*n* = 346), which required at least two secure International Classification of Diseases (10th edition, ICD-10) diagnoses G35 and a recorded visit with a neurologist, and three control cohorts, including children newly diagnosed with Crohn’s disease (*n* = 2510), psoriasis (*n* = 14,234), and a group without these autoimmune diseases (No AID) (*n* = 1645) served as controls.^7^ The No AID cohort was matched to the MS cohort in approximately 1:3 ratio based on birth year, sex, and district of residence. All participants were aged 9–17 years.

In a sensitivity analysis, we excluded persons with ICD codes of diseases that may reflect symptoms of undiagnosed MS in the 5 years before the diagnosis (Supplementary Table 1).

We conducted another analysis on a subset of patients from the main cohort who were diagnosed with secured ICD G04 (encephalitis, myelitis, encephalomyelitis, or clinically isolated syndrome (CIS)), suspected ICD G35 (multiple sclerosis, not secured), and secured ICD H46 (optic neuritis) (Supplementary Table 1). This analysis, termed the CIS analysis, focused specifically on the 5 years preceding the first neurological symptoms in patients who were later diagnosed with MS.

We present the distribution of any vaccination over the entire 5 years in both absolute and relative frequencies. We used multiple logistic regression models to investigate the association between pediatric MS and vaccinations the 5 years before diagnosis of MS or CIS. These models used MS (yes/no) as a binary outcome contrasting this cohort against each of the other cohorts, using vaccinations administered (yes = at least one/no = never) as an independent variable and further adjustment for sex and age. Results are reported using odd ratios (ORs) and corresponding 95% confidence intervals (CIs). In addition, we used Poisson regression models to compare the frequency of vaccination between the cohorts.

Hypothesis testing was performed at exploratory two-sided 5% significance levels. All analyses were conducted using R 4.4.0.

## Results

We found that children with MS had lower vaccination rates (65.3%) compared to those with No AID (68.4%) in the 5 years before diagnosis (Table 1 and Supplementary Tables 1 and 2). Over the 5 years before diagnosis, the age and sex standardized relative frequencies also showed a lower vaccination in children with MS compared to No AID (68.8%), Crohn’s disease (69.0%), and psoriasis (67.4%). The annual age and sex standardized relative frequencies also indicate a lower vaccination in children with MS (Supplementary Figure 1).

**Table 1.** Descriptive statistics of the cohorts and vaccinations.

	MS	No AID	Crohn’s disease	Psoriasis
Main analysis	*n* (%)	*n* (%)	*n* (%)	*n* (%)
**Size**	346	1645	2510	14,234
**Female**	260 (75.1)	1208 (73.4)	1240 (49.4)	7775 (54.6)
**Age, mean** **±** **SD**	15.6 **±** 1.6	15.0 ± 1.6	14.3 ± 2.4	13.4 (2.5)
**Any vaccination**	226 (65.3)	1125 (68.4)	1694 (67.5)	9715 (68.3)
**Standardized relative frequency of any vaccination**	65.3	68.8	69.0	67.4
**Sensitivity analysis**				
**Size**	122	960	1378	8023
**Female**	89 (73)	703 (73.2)	642 (46.6)	4292 (53.5)
**Age, mean** **±** **SD**	15.7 **±** 1.4	15.1 **±** 1.5	14.5 **±** 2.3	13.7 **±** 2.5
**Any vaccination**	65 (53.3)	623 (64.9)	866 (62.8)	5155 (64.3)
**Standardized relative frequency of any vaccination**	53.3	65.1	64.7	64.4
**CIS analysis**				
**Size**	72	1645	2510	14,234
**Female**	62 (86.1)	1208 (73.4)	1240 (49.4)	7775 (54.6)
**Age, mean** **±** **SD**	15.4 **±** 1.7	15.0 **±** 1.6	14.3 **±** 2.4	13.4 **±** 2.5
**Any vaccination**	49 (68.1)	1125 (68.4)	1694 (67.5)	9715 (68.3)
**Standardized relative frequency of any vaccination**	68.1	70.7	69.8	69.0

**Figure 1.**
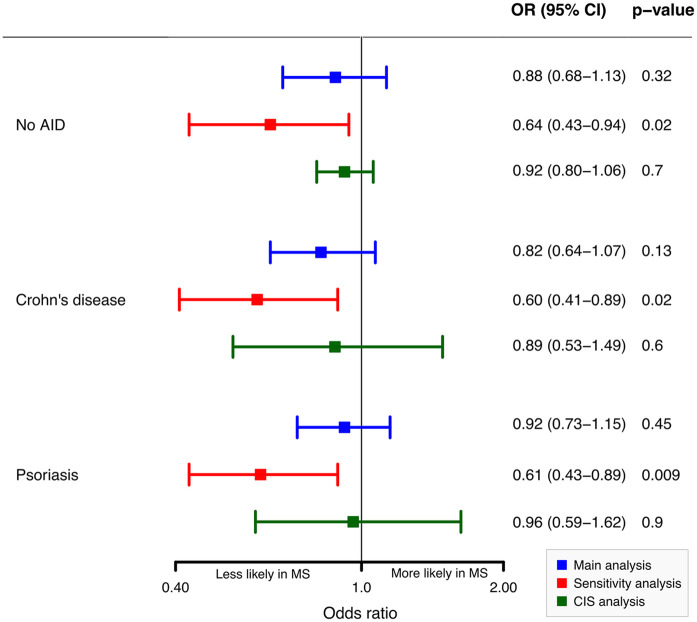
Odd ratios (ORs) of any vaccination for MS in the 5 years before the diagnosis.

The multiple analysis by logistic regression models revealed negative and non-significant associations between vaccination records within the 5 years preceding diagnosis and the odds of diagnosing pediatric MS. Compared to children with No AID, the OR was 0.88 (*p* = 0.32). Compared to children with Crohn’s disease, the OR was 0.82 (*p* = 0.13); compared to children with psoriasis, the OR was 0.92 (*p* = 0.45) (Figure 1).

The negative associations increased in a sensitivity analysis excluding children with ICD-10 codes that might reflect undiagnosed MS. In the CIS analysis, associations remained consistent (Figure 1). The Poisson regression model shows minor negative associations between MS diagnosis and vaccination counts compared to the No AID cohort (IRR = 0.96, 95% CI = 0.84–1.11), Crohn’s disease cohort (IRR = 0.97, 95% CI = 0.84–1.11), and psoriasis cohort (Incidence Rate Ratios (IRR) = 0.99, 95% CI = 0.86–1.12). Vaccination counts in the MS cohort were slightly lower compared to other cohorts (Supplementary Table 3).

## Discussion

In Germany, a comprehensive childhood vaccination schedule starting at 6 weeks of age, covering vaccines for rotavirus, DPTPH (diphtheria, pertussis, tetanus, polio, and *Haemophilus influenzae* type B), hepatitis B, pneumococcal infections, MMRV (measles, mumps, rubella, and varicella), meningococcal infections, and HPV is recommended. The majority of booster vaccinations are administered between the ages of 9 and 16, including those for DPTPH, hepatitis B, pneumococcal infections, meningococcal infections, measles, and HPV.^8^

Our findings do not indicate that childhood vaccination in the 5 years before the diagnosis is associated with an increased risk of pediatric MS. This conclusion aligns with previous studies that have found no significant link between vaccinations and the onset of MS or other autoimmune diseases. The results of a recent study from our group indicate that vaccination is also not a risk factor for MS in adults.^7^ Similarly, a study in France showed that vaccination against the hepatitis B virus does not increase the risk of pediatric MS.^9^

The implications of these findings are substantial for public health. Vaccinations are foundational to public health strategies, effectively preventing the transmission of infectious diseases and their severe outcomes.^10,11^ The negative and non-significant association observed in our study underscores the importance of maintaining trust in childhood vaccination programs.

This is the first study to cover more than 300 children with pediatric MS, including all vaccinations administered in the 5 years before diagnosis. However, it is crucial to acknowledge the limitations of our study, including potential biases related to coding practices and data completeness inherent in claims data, as well as the retrospective design, which limits causal inferences. Although vaccinations are free of charge all over Germany, we do not have information about the socioeconomic status of participants, which could influence other health-related behaviors or access to healthcare services, potentially confounding the results. In addition, our study only covered the 5 years before diagnosis, which precludes any assumptions on the effect of vaccinations more than 5 years before diagnosis. Future research should prioritize prospective cohort studies and larger population-based studies to validate our findings and further explore this association.

## Conclusion

Our study findings do not indicate that vaccination during childhood is associated with an increased risk of pediatric MS in the 5 years preceding diagnosis. Vaccinations remain a critical component of public health strategies, effectively preventing the transmission of infectious diseases and their severe outcomes. These results underscore the importance of maintaining trust in childhood vaccination programs.

## Supplemental Material

sj-docx-1-msj-10.1177_13524585241297003 – Supplemental material for Vaccination as a risk factor for pediatric multiple sclerosis: Insights from a retrospective case–control study Supplemental material, sj-docx-1-msj-10.1177_13524585241297003 for Vaccination as a risk factor for pediatric multiple sclerosis: Insights from a retrospective case–control study by Sonia Darvishi, Ewan Donnachie, Christiane Gasperi, Alexander Hapfelmeier and Bernhard Hemmer in Multiple Sclerosis Journal
