# Supplementary material for: Vaccination as a risk factor for pediatric multiple sclerosis: Insights from a retrospective case–control study
Source: Mult Scler. 2024 Nov 14;30(14):1825–9. doi: 10.1177/13524585241297003 (PMC11616212; doi:10.1177/13524585241297003)
Supplement: sj-docx-1-msj-10.1177_13524585241297003 – Supplemental material for Vaccination as a risk factor for pediatric multiple sclerosis: Insights from a retrospective case–control study [file sj-docx-1-msj-10.1177_13524585241297003.docx]

**Supplementary 1**

In the sensitivity analysis, we excluded persons with record of vision problems (H53), cranial nerve disorder (G35), disease of vestibular function (H81), neuromuscular dysfunction of bladder (N31), disturbances of skin sensation (R20), abnormalities of gait and mobility (R26), unspecified urinary incontinence (R32), signs involving the genitourinary system (R39), and dizziness and staggering (R42).

**Supplementary 2**

In the CIS analysis, we included persons in the MS cohort with a record of G04 (encephalitis, myelitis, encephalomyelitis, or clinically isolated syndrome (CIS)), G35 (cranial nerve disorder), and H46 (optic neuritis) in the 5 years before the diagnosis.

**Supplementary 3**

We used Poisson regression models to compare the frequency of vaccination between the cohorts. These models were structured similarly to the logistic regression models and were used to estimate incidence rate ratios (IRR) and marginal means with 95% confidence intervals, adjusted for sex and age.

**Supplementary Fig. 1** **Standardized^*^ relative frequency of any vaccination usage 5 years before diagnosis.**


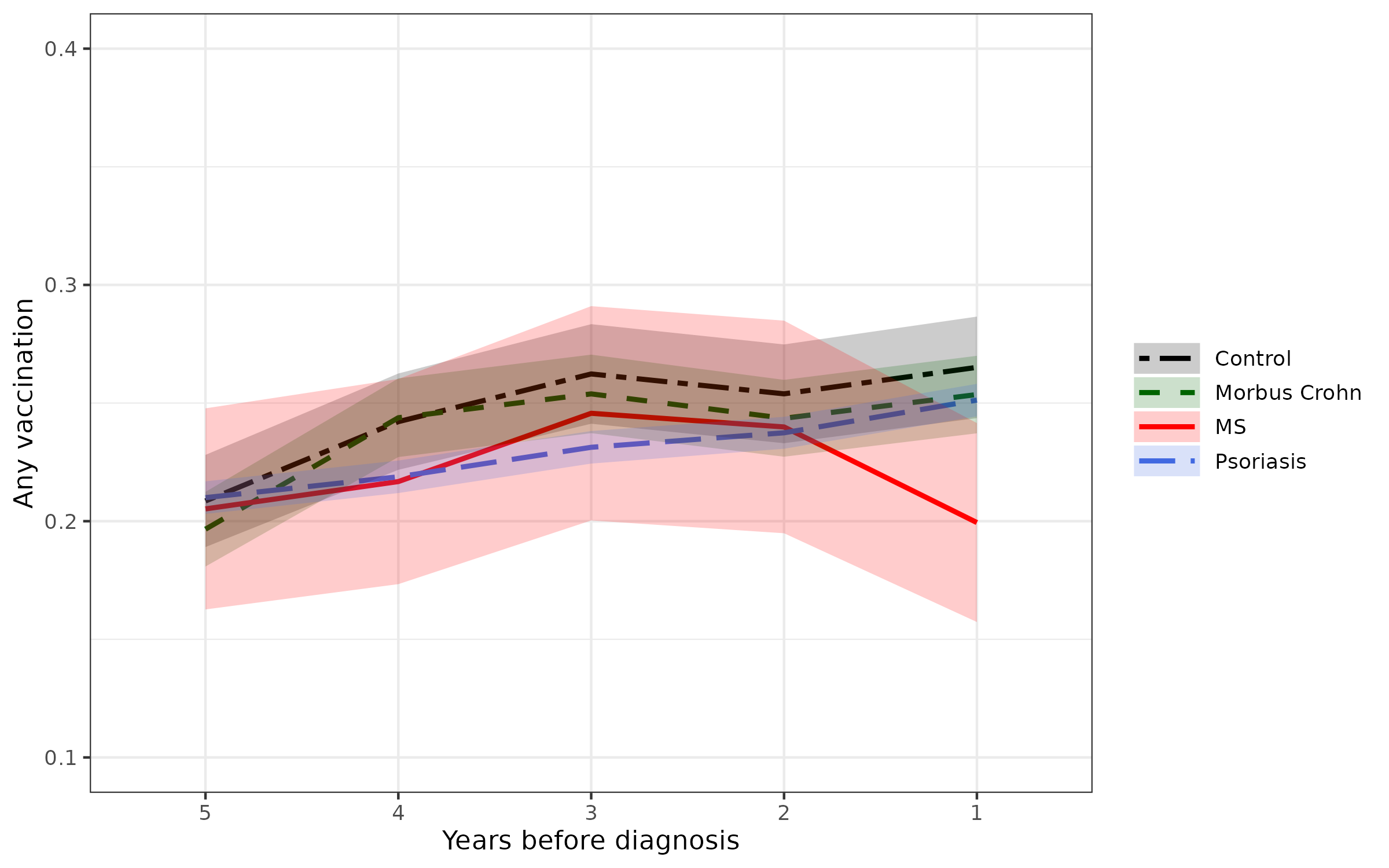


*The relative frequencies were standardized to the age and sex distribution of the MS cohort.

**Supplementary Table 1 Incidence rate ratios (IRR) in the 5 years before diagnosis for Pediatric MS vs. control cohorts.**

|  | **Predictor** | **Mean^a^ count (95% CI)** | **IRR** | **95% CI** | **p-value** |
| --- | --- | --- | --- | --- | --- |
| **Any vaccination** |  |  |  |  |  |
|  | MS | 0.70 (0.61-0.80) | - | - | - |
|  | No AID | 0.72 (0.68-0.76) | 0.96 | (0.84, 1.11) | 0.66 |
|  | Crohn’s disease | 0.72 (0.69-0.75) | 0.97 | (0.84, 1.11) | 0.69 |
|  | Psoriasis | 0.71 (0.96-0.72) | 0.99 | (0.86, 1.12) | 0.87 |
| ^a^Marginal means adjusted for age and sex | | | | | |
